# Editorial: Emerging Challenges in the Diagnosis and Treatment of Autoimmune Encephalitis

**DOI:** 10.3389/fneur.2019.00146

**Published:** 2019-02-25

**Authors:** Morten Blaabjerg, Thomas Seifert-Held, Johann Sellner

**Affiliations:** ^1^Department of Neurology, Odense University Hospital, Odense, Denmark; ^2^Department of Clinical Research, University of Southern Denmark, Odense, Denmark; ^3^Department of Neurology, Medical University of Graz, Graz, Austria; ^4^Department of Neurology, Christian Doppler Medical Center, Paracelsus Medical University, Salzburg, Austria; ^5^Department of Neurology, Klinikum Rechts der Isar, Technische Universität München, München, Germany

**Keywords:** autoimmune, encephalitis, diagnosis, therapy, outcome, challenges

Autoimmune encephalitis (AIE) is a group of antibody-mediated inflammatory CNS diseases with a variety of neurological and psychiatric symptoms. Patients often have deficits of subacute onset with affection of both limbic (e.g., amnesia, confusion, and epileptic seizures), and extra-limbic brain structures (1). Previously, AIE was considered a very rare paraneoplastic condition associated with intracellular (onconeural) antibodies and a very poor prognosis (e.g., anti-Hu syndrome) (2). However, during the last two decades it has become evident that AIE is much more frequently associated with antibodies directed against synaptic/cell surface proteins and often an underlying malignancy is absent. More than 10 synaptic antineuronal and glial antibodies associated with AIE have been identified, and new antibodies are described at an astonishing pace; many patients with so-called seronegative AIE will probably harbor antibodies that are yet to be isolated. Prognosis in AIE can be good, if aggressive immunomodulatory treatment is initiated early (3). Yet, chronic cognitive deficits, epilepsy and mood disorders are frequent, and this is particularly concerning in young patients who frequently are unable to join the workforce again (4). Thus, in addition to significant health-related concerns for the individual, AIE is also associated with substantial socioeconomic burden.

In the 11 articles that form this Frontiers Research Topic, eBook, the readers will find an update on key aspects including diagnostic challenges, pitfalls in antibody testing and clinical experience with management and treatment of AIE and related disorders from different expert centers.

Firstly, the current syndromes associated with antibodies against cell surface antigens, including the use of the current diagnostic criteria and treatment options are presented in a mini review paper by Hermetter et al. This is followed with real life clinical experience in a monocentric study of 38 consecutive patients with AIE presented by Macher et al. In this paper the important aspect of when to stop immunotherapy after the acute phase of encephalitis is also discussed based on their clinical experience.

One of the most important aspects in the diagnosis of AIE is identification of autoantibodies in CSF and/or serum. This is currently done using different antibody assays. The pitfalls of antibody detection and the use of different assays (e.g., cell or tissue based assays, radioimmunoassay) is elegantly reviewed by Ricken et al. This paper highlight the current state of the art in antibody testing dependent on antibody subtype and provides important consideration on assay choice and interpretation. The potential pitsfalls in antibody testing is further highlighted in a case description by Bien, presenting a case of overinterpretation and overtreatment of a patient with a low-titer contactin-associated protein-like 2 (Caspr2) antibody. Moreover, an attempt to optimize antibody detection is provided by Chiu et al. who present changes to the conventional anti-NMDAR antibody assay in order to increase detection rates.

Infectious encephalitis (IE) is one of the main differential diagnoses in the acute phase of AIE and due to the need for early treatment initiation, discrimination between these conditions are crucial. In their paper, Wagner et al. included all patients from their center with diagnosis of encephalitis over a 10-year period (33 AIE, 51 IE), and present some interesting distinctive clinical features (e.g., headache, fever, psychiatric symptoms). Applying the current diagnostic criteria for AIE to this cohort however, yielded very low diagnostic sensitivity and specificity. They moreover found this phenomenon to be clearly time dependent.

An association between demyelinating diseases and AIE has been established. The paper by Borisow et al. reviews these diseases and gives an overview on the diagnosis and treatment of neuromyelitis optica spectrum disorders (NMOSD) and the more recently described myelin-oligodendrocyte-glycoprotein (MOG)-associated encephalomyelitis. The paper highlights the differences in epidemiology, imaging and provides current knowledge on treatment strategy.

As the field of AIE expand, clinical descriptions of cases that differ from the original published case series are important to expand knowledge on the different antibody specific phenotypes. Two such cases are presented in this ebook. Montagna et al. describe a case of IgLON5-associated encephalitis with severe inflammatory lesions on brain MRI and no tau pathology on brain biopsy. The presented patient moreover had an atypical presentation with a rapid cognitive decline and a good response to immunotherapy. Interestingly, they found antibodies exclusively of the IgG1 subclass and not the prodominant IgG4 subclass initially described with IgLON5 encephalitis.

Similarly Bartels et al. describe three cases of Anti-ARCHGAP26 (RhoGTPase-activating protein 26) antibodies in whom, two were associated with isolated cognitive impairment and not with the cerebellar ataxia phenotype usually associated with this antibody. All three cases were found to have underlying malignancy.

Besides the general treatment recommendations described above, two further papers deals with this aspect. The paper by Mäkelä et al. focuses on the difficult clinical management of epilepsy associated with the Glutamic acid decarboxylase (GAD65) antibody. They present 6 cases from their center treated between 2013 and 2017, and highlight the importance of early immunotherapy in order to prevent tissue damage and refractory epilepsy in this patient group.

Finally, Zhu et al. performed an experimental interleukin-27 gene therapy using adeno-associated viral vector delivery to an experimental model of inflammatory disease. They were able to prevent the development of experimental autoimmun encephalomyelitis (EAE) in this model, but this approach was not effective in established inflammation likely due to expansion of myeloid cells.

In summary, although the field is rapidly expanding, we believe that the present Frontiers Research Topic eBook will provide the interested readers with updated knowledge on AIE including real life clinical experience in diagnostic challenges, differential diagnosis and treatment of patients with AIE.

## Author Contributions

All authors listed have made a substantial, direct and intellectual contribution to the work, and approved it for publication.

### Conflict of Interest Statement

The authors declare that the research was conducted in the absence of any commercial or financial relationships that could be construed as a potential conflict of interest.
